# Acceptance Factors for In-Hospital Pharmacist Interventions in Daily Practice: A Retrospective Study

**DOI:** 10.3389/fphar.2022.811289

**Published:** 2022-03-23

**Authors:** Amaury Durand, André Gillibert, Sophie Membre, Lisa Mondet, Aurélie Lenglet, Aurélien Mary

**Affiliations:** ^1^ Department of Pharmacy, Amiens-Picardie Teaching Hospital, Amiens, France; ^2^ Department of Pharmacy, Intercommunal Hospital of the Baie de Somme, Saint Valery sur Somme, France; ^3^ Department of Biostatistics, Rouen Teaching Hospital, Rouen, France

**Keywords:** clinical pharmacy, quality of care, contact method, pharmacist interventions, medication review

## Abstract

**Introduction:** Performing pharmacist interventions (PIs) during the medication review helps to improve the quality of care. The acceptance by the physician of these PIs is a good indicator of the quality of this clinical pharmacy activity. The objective of this study was to determine, in the Amiens-Picardie teaching hospital (France), factors of acceptance in a variable environment of activity (central pharmacy, in the care units, computer assisted).

**Methods:** All PIs transcribed by pharmacists on the Act-IP© site between November 2018 and April 2019 were analyzed using a complementary search in patient records. The environment, type, and clinical impact on patient health of each PI was collected. Linear mixed-effects models with a random pharmacist intercept were used to investigate the relationship between PI modalities and their chance of being accepted.

**Results:** A total of 3,100 PIs were traced, of which 2,930 had been followed over time. Of these, 2,930 PIs, 1,504 (51.3%) were performed by a postgraduate pharmacist and 1,426 (48.7%) by a pharmacy resident, 1,623 (55.4%) were performed by verbal exchange, 455 (15.5%) by telephone, 846 (28.9%) by computer software, and 6 (0.2%) by paper. The clinical impact on patient health was major for 976 PIs (33.3%) and vital for 26 PIs (0.9%). According to the Anatomical Therapeutic Chemical Classification (ATC), they were mainly related to anti-infectives (30.3%), the nervous system (18.7%), and blood and blood-forming organs (17.3%). In total, 2,415 PIs (82.4%) were accepted. According to the multivariate model, a PI was more often accepted when it was transmitted orally rather than by software (+27.7%, 95% CI: +23.2 to +32.1%) and when it was transmitted to a medical resident rather than a postgraduate physician (+4.4%, 95% CI: 1.2–7.6%). In these cases, there was a major rather than a moderate clinical impact on patient health (+4.3%, 95% CI: +1.1–+7.6%).

**Conclusion:** This study highlights the importance of the quality of the exchange with the prescriber and the prioritization of high-risk interventions as key points of medication review to improve rate of pharmacist interventions accepted by physician.

## Introduction

Adverse drug reactions are responsible for approximately 5.3% of hospitalizations in the general population, with a higher prevalence in the geriatric population (Kongkaew et al., 2008). Medication-related hospital admissions are preventable in 68% of cases (Zed et al., 2008). In hospitals, adverse drug reactions are preventable in 37.3% of cases and lead to longer hospital stays and higher health care costs (Formica et al., 2018).

Clinical pharmacy activities such as training caregivers, creating drug use protocols, participating in medical visits, managing drug reconciliation, or medication review favoring proper use and optimization of prescribing reduce the risk of adverse drug reactions and are associated with lower hospital mortality (Bond and Raehl, 2007) and lower health care costs (Bond and Raehl, 2006). In the OPTIMIST multicenter randomized study, an extended PI based on medication review, motivational interview, and follow-up after discharge reduces the readmission rate to 30 and 180 days, from 22.3 to 14.3%, and from 48.8 to 39.7%, respectively (Ravn-Nielsen et al., 2018). The role of the pharmacist in the correct use of medicines is recognized in various medical specialties, such as geriatrics (Martin et al., 2018), infectious disease (Livorsi et al., 2020), intensive care (Lee et al., 2019), surgery (Neville et al., 2014), and cardiology (Zhai et al., 2016).

Medication review leading to PIs can be performed with varying degrees of comprehensiveness depending on the patient (care unit, general condition) and pharmacist (experience, available resources) parameters. It is a structured evaluation of a patient’s medicines with the aim of optimizing the use of medicines and improving health outcomes (Griese-Mammen et al., 2018). It can range from a simple drug-drug interaction analysis, for example, at the time of dispensing, to a personalized medication plan (medication review and motivational interview in collaboration with community health professionals) (Allenet et al., 2019). Regardless of the number and clinical impact of the PIs transmitted to physicians, they are only of value if they are followed by a change in prescription.

Due to financial and human resource constraints, medication review (when performed) is most often done from the hospital pharmacy, with limited time and access to medical data with high heterogeneity between hospitals. In France, the ratio of hospital pharmacists to hospital beds is quite low compared to Anglo-Saxon countries (such as Australia), resulting in limited pharmaceutical care activity dedicated to patients (Roulet et al., 2014; Weier et al., 2018; Pourrat et al., 2020). In Canada, the United States, and Australia the priority for increasing the efficiency of clinical pharmacy activity in hospitals has been the assignment of clinical pharmacists directly to the wards (Rose et al., 2018). These differences reflect a lack of confidence in clinical pharmacy activity among European health economists (Garattini et al., 2021). This highlights the constant need to reassure health managers about the performance of clinical pharmacy.

At the Amiens-Picardie teaching hospital (France), medication review can be performed in different settings: 1) a within-the-pharmacy review, a level 2B analysis with access to laboratory findings and computerized patient records, but without seeing the patient; 2) a within-the-ward review with pharmacists physically in a clinical unit (a level 3 analysis, including patient visits) (Saint-Germain et al., 2016; Mary et al., 2019), 3) an alternative within-the-ward review through mobile teams, with pharmacists accompanying specialized physicians to get medical advice on a specific request [either an infectious disease team (Mabille et al., 2020) or a geriatrics team]; or 4) a review with a medical decision support system (MDSS) Pharmaclass ® (Membre et al., 2019). Pharmaclass^®^ is a computer application that generates alerts to target patients who may need a medication review. The alert, generated by the MDSS, is based on rules that cross-reference prescription data with laboratory and clinical data on the patient (e.g., prescription of direct oral anticoagulants and a GFR<15 ml/min). The rules are coded by pharmacists and present a course of action to assist the pharmacist. The rules coded over the study period mainly targeted anticoagulants and are described elsewhere (Membre et al., 2019).

The objective of the present study was to evaluate, in this institution, the factors favoring acceptance of PIs by prescribers, with particular emphasis on the modality of the activity performed by the pharmacist and the clinical impact on patient health of PIs.

## Materials and Methods

A retrospective, single-center study was conducted in a 1,673-bed teaching hospital (Amiens-Picardie teaching hospital; France). The study was approved and registered by Amiens-Picardie teaching hospital’s local research department under the number PI2022_843_0008. Postgraduate pharmacists and pharmacy residents analyzed computerized medical prescriptions on the hospital prescription assistance software DxCare™ (Medasys, Le Plessis-Robinson, France, version 7.7.2) or Clinisoft™ (for intensive care units) (GE Healtcare, Barrington, Illinois, United States, version 7.0). In France, pharmacy or medical residents are students who have already completed at least 5 (pharmacy residency) or 6 (medical residency) years of study and who perform many of the tasks assigned to postgraduate pharmacists and postgraduate physicians. They are under the responsibility of a postgraduate and have not yet attained the status of a PharmD or M.D., which is obtained after a minimum of 3 years of residency in combination with a postgraduate diploma, “diplôme d’études supérieures (DES)”. Prescriptions were analyzed either within the pharmacy or within the wards for surgical resuscitation (16 beds), hematology (30 beds), medical oncology (17 beds), orthopedic surgery (38 beds), acute geriatrics (60 beds) and post-acute rehabilitation (37 beds). Some pharmacists with ward responsibility could also perform within-the-pharmacy medication reviews (for example, during on-call duty or while periodically cross-analyzing, targeting at-risk drugs). Moreover, a within-the-pharmacy medication review could sometimes involve wards where a pharmacist in their team is present (for example, during nominative drug dispensing, during on-call duty, or pharmacist vacation). In cases of potentially inappropriate prescriptions, the pharmacist first transmits the PI to the physician, then document the PI and its outcome on the Act-IP© website [French Society of Clinical Pharmacy (SFPC), Marseille, France, version 2]. Act-IP© is a website that allows users to register and extract PIs which are standardized with SFPC codification (Allenet et al., 2006). The process follows the recommendations of the SFPC for daily activity (Allenet et al., 2006) and are reinforced by an internal quality procedure that insists on archiving in Act-IP©. In order to harmonize the practice, each new pharmacist is trained in the procedure, and clinical pharmacy meetings are held every month. The acceptance rate is not transmitted during these interventions, which avoids an indicator optimization bias. All PIs transcribed on Act-IP© between November 2018 and April 2019 were extracted and analyzed.

The following variables were extracted from the Act-IP© database:

• Patient age.
• Patient gender.
• Care unit.
• Status of the pharmacist (postgraduate or resident) performing the PI.
• Location in which the PI was performed (pharmacy or care unit).
• Status of the prescriber (postgraduate or resident).
• Contact method of the PI.
• Anatomical Therapeutic Chemical (ATC) drug class concerned by the PI.
• Problem encountered according to the SFPC coding system (Allenet et al., 2006).
• Type of PI according to the SFPC coding system (Allenet et al., 2006).
• CLinical, economic, and organizational (CLEO) impact of the PIs according to the recently published CLEO scale (Vo et al., 2019). Briefly the clinical dimension reflects the risk of complication avoided by the PI and is coded on a Likert scale (from −1C: negative PIs to 4C: vital PIs). A PI is considered to have a major clinical impact on patient health when it avoids a risk of prolonged hospitalization, a permanent disability or handicap. The economic and organizational impact are coded on a 3-point Likert scale (from −1 to +1).
• Use, or not, of the medical decision support system (MDSS) Pharmaclass®.
• Pharmacist intervention carried out or not following the reconciliation of drug treatment.
• Outcome of PI (accepted or not by the physician).

An independent complementary documentation was performed for all PIs whose acceptance was recorded as unknown to complete missing data on that variable, based on the actual modification of the prescription.

### Outcome

The primary outcome was the acceptance of the PI by the physician, in binary form (yes/no).

### Statistical Analysis

The association between acceptance rate and covariables was studied by Gaussian linear mixed-effects models with a random pharmacist intercept. The linear mixed effect, without transformation, was chosen to allow expression of the results as absolute percentage differences rather than odds ratios. The sample size was sufficient for the Gaussian approximation to be valid.

All statistical analyses were performed with R software (version 4.0, The R Foundation for Statistical Computing, Vienna, Austria) (Davis, 2010). Because of the exploratory nature of the work and to maintain reasonable statistical power, no multiple testing procedure were done, but a two-sided significance level at 1% was chosen.

The main multivariate analysis was carried out using a model explaining the acceptance of the PI by almost all the parameters collected on Act-IP© (age, sex, hospital ward, status of the pharmacist, status of the prescriber, method of contact, ATC class, problem encountered, type of intervention, clinical, organizational and economic impact, PI during reconciliation, use of Pharmaclass®), assuming that all these parameters were likely to influence the chances of acceptance. Only the location in which the PI was performed was not included in the model of the primary analysis because it was too strongly correlated with the method of contact of PIs, almost exclusively by verbal exchange in the care units. This main analysis was completed by minimally adjusted analyses, in models explaining the chances of PI acceptance by each of the covariates (one at a time) while retaining a random pharmacist effect. A subgroup analysis by the same statistical models was then performed in the subgroup of PIs with major or vital clinical impact on patient health.

*Post hoc* analyses were performed, specifically looking at the effect of the location in which the PI was performed, in crude analysis (Chi^2^ test), minimally adjusted (mixed-effects model with random pharmacist effect) and adjusted for the contact method of the PI with random pharmacist effect.

For three variables, it was not possible to choose a reference modality without it strongly affecting the interpretation of the results: the ATC class of the drug concerned, the type of potentially inappropriate prescription (drug not indicated, drug interaction, underdosing, etc.), and the recommendation (addition, discontinuation, dosage adjustment, etc.). These variables were contrast coded by a diagram known as “effects coding” or “sum to zero contrasts” as implemented by the contr. sum function in R software. This coding provides, as a reference modality, the average of the effects of all the modalities of the variable. Each effect is then expressed as the difference between the effect of the modality of interest and the average of the effects of all the modalities of the variable. By mathematical construction, the sum of all the effects of all the categories of a variable is always zero with this coding. A *post hoc* analysis was performed by recoding the ordinal categorical clinical impact on patient health variable as a discrete quantitative variable (0 = no impact, 1 = minor, 2 = moderate, 3 = major, 4 = vital) in order to assess a linear trend in the acceptance rate.

### Missing Data

Pharmacist interventions with unknown acceptance status (yes/no) were excluded from all analyses. There was enough missing data on the primary outcome to test the hypothesis of differential reporting bias by contact modality (software, telephone, verbal), using a Chi^2^ test on the rate of missing data by contact modality. Missing data on the contact modality (*n* = 32) were simply imputed as a random value of the same variable. Then, missing data for clinical (*n* = 299), economic (*n* = 416), and organizational (*n* = 347) impacts were simply imputed as a random value of the same variable chosen within the same contact modality.

## Results

### Descriptive Presentation of Results

From November 2018 to April 2019, 3,100 PIs were recorded on Act-IP^©^ by 24 pharmacists. After exclusion of PIs with missing acceptance status (*n* = 170), 2 930 PIs remained (Table 1).

**TABLE 1:** Descriptive presentation of PIs performed with acceptance status entered.

Pharmacist	
Postgraduate pharmacist	1,426 (48.7%)
Pharmacy resident	1,504 (51.3%)
Prescriber	
Postgraduate physician	802 (27.4%)
Medical resident	2,128 (72.6%)
Patient gender	
Male	1,421 (48.5%)
Female	1,509 (51.5%)
Patient age	
≤59	773 (26.4%)
60–69	595 (20.3%)
70–79	569 (19.4%)
80–89	682 (23.3%)
≥90 years	311 (10.6%)
Problem encountered	
Non-compliance with guidelines and contraindications	687 (23.4%)
Untreated indication	329 (11.2%)
Underdosing	392 (13.4%)
Overdosing	559 (19.1%)
Drug not indicated	203 (6.9%)
Drug interaction	135 (4.6%)
Adverse effect	57 (1.9%)
Inappropriate route and/or administration	267 (9.1%)
Treatment not received	2 (0.1%)
Monitoring to be followed	299 (10.2%)
Type of intervention	
Addition	413 (14.1%)
Discontinuation	497 (17%)
Substitution/Exchange	358 (12.2%)
Choice of route of administration	14 (0.5%)
Therapeutic follow-up	385 (13.1%)
Optimization of administration modalities	255 (8.7%)
Dosage adjustment	1,008 (34.4%)
Method of contact	
Verbal	1,623 (55.4%)
Phone call	455 (15.5%)
Prescription assistance software	846 (28.9%)
Paper	6 (0.2%)
Clinical impact on patient health	
None	41 (1.4%)
Minor	570 (19.5%)
Moderate	1,317 (44.9%)
Major	976 (33.3%)
Vital	26 (0.9%)
Organizational impact	
Unfavorable	89 (3%)
Nil	2,130 (72.7%)
Favorable	711 (24.3%)
Economic impact	
Unfavorable	788 (26.9%)
Nil	1,172 (40%)
Favorable	970 (33.1%)
ATC class	
A Digestive tract and metabolism	409 (14%)
B Blood and blood-forming organs	508 (17.3%)
C Cardiovascular system	272 (9.3%)
D Dermatological drugs	8 (0.3%)
G Genitourinary system and sex hormones	33 (1.1%)
H Systemic hormones, excluding sex hormones	74 (2.5%)
J General anti-infectives for systemic use	887 (30.3%)
L Antineoplastics and immunomodulators	68 (2.3%)
M Muscle and skeleton	37 (1.3%)
N Nervous system	547 (18.7%)
P Antiparasitic insecticides	7 (0.2%)
R Respiratory system	44 (1.5%)
S Sensory organs	14 (0.5%)
V Miscellaneous	19 (0.6%)
Z Drug with no code assigned	3 (0.1%)
PI at reconciliation	
No	2,586 (88.3%)
Yes	344 (11.7%)
PI using Pharmaclass®	
No	2,786 (95.1%)
Yes	144 (4.9%)
Validation setting	
Within the pharmacy	1,096 (37.4%)
Within the ward	1,834 (62.6%)
Care unit	
Nursing home	282 (9.6%)
Medicine-surgery-obstetrics departments, excluding resuscitation	1,931 (65.9%)
Resuscitation	717 (24.5%)
Number of drugs per PI	
1	2,349 (80.2%)
2	499 (17%)
3	51 (1.7%)
4	31 (1.1%)

Almost twice as many PIs were performed by pharmacists within the wards compared to those within the pharmacy. About three-quarters of PIs were communicated to medical residents. A minority (5%) were performed through the Pharmaclass™ tool and 11.7%% during medication reconciliation. The three main problems encountered were non-compliance with guidelines and contraindications (23.4%), overdosing (19.1%), and underdosing (13.4%). The three main PI recommendations were requests for dose adjustment (34.4%), discontinuation (17.0%), and addition (14.1%). The clinical impact of PIs was most often estimated to be moderate or major for one-half and one-third of PIs, respectively.

### Acceptance Rate of Pharmacist Interventions

A total of 2,415 PIs (77.9%) were traced as accepted and 515 (16.6%) as refused, while the acceptance status of 170 (5.5%) pIs could not be found despite retrospective analysis of patient records. Under the maximum bias assumption, the acceptance rate ranged from 77.9%, considering missing data as refusals, to 83.4%, considering missing data as acceptances. There was a significant difference (*p* < 0.0001 by Chi^2^ test) in the rate of missing data on PI acceptance according to the method of contacting the prescriber, with 2.1% (35/1,658) missing data in cases of verbal exchange, 3.0% (14/469) by telephone, 12.4% (120/966) by the prescriber assistance software, 0% (0/6) by paper, and 100% (1/1) by e-mail. Subsequent statistical analyses excluded observations with missing data on PI acceptance status, and thus 2,930 PIs were retained. After excluding these missing data, the acceptance rate was estimated to be 82.4% (2,415/2,930).

### Description by Practice Setting

The acceptance rate of PIs by physicians performed by pharmacists practicing on the wards varied: 98.0% in hematology (96/98), 97.9% in surgical resuscitation (599/613), 97.5% in orthopedic surgery (344/353), 86.7% in oncology (247/285), and 67.1% in acute geriatrics (282/420). Overall, the acceptance rate of PIs was significantly higher when the analysis was performed within the wards (89.0%, 1,633/1,834) rather than from the pharmacy (71.4%, 782/1,096) (*p* < 0.001 by Chi^2^ univariate analysis).

### Description of Pharmacist’s Activities

A total of 24 pharmacists performed the 2,930 PIs, with a median of 22 PIs per pharmacist (interquartile range: 11–256), with a maximum of 503 and a minimum of 1. Three pharmacists had performed 46.9% of the PIs (1 374/2 930). The six pharmacists with the highest activity had performed 80.1% (2,348/2,930) of the PIs and eight pharmacists had performed 92.3% (2,703/2,930) of the PIs. Of these 24 pharmacists, 18 exclusively performed within-the-pharmacy medication review, four performed almost exclusively within-the-ward medication review (≥94%), and two performed mostly within-the-ward medication review (78.4 and 83.5%). In the absence of statistical adjustment (including within-the-ward/pharmacy settings), the inter-pharmacist standard deviation of the acceptance rate was estimated at 18.7% (absolute). In the main analysis model adjusted for all variables, the inter-pharmacist standard deviation of the acceptance rate was estimated at 12.6% (absolute). Thus, some of the inter-pharmacist variance was attributable to differences in the setting in which the PIs were performed.

### Determination of Factors Associated With Accepted Pharmacist Interventions, Imputed Modality Data

Among the 2,930 PIs analyzed (accepted or refused), 32 (1.1%) method of contact data were not entered; and they were simply imputed by a random value of the same variable. The clinical impact on patient health was missing for 299 (10.2%) PIs, the organizational impact for 347 (11.8%) PIs, and the economic impact for 416 (14.2%) PIs. They were simply imputed by a random value of the same variable in the corresponding contact modality.

According to the minimally adjusted statistical analysis (pharmacist random effect) on the 2,930 PIs, the factors significantly associated with PI acceptance were: PI performed orally (+30.0% acceptance vs. software, *p* < 0.0001), major (+5.1% vs. mean, *p* = 0.001) or minor (−6.7% vs. mean, *p* < 0.0001) clinical impact, resident prescriber (+5.4%, *p* = 0.0008), and PI during reconciliation (+5.7%, *p =* 0.008).

In the adjusted multivariate statistical analysis on all parameters (main analysis), factors associated with accepted PIs were: PI performed orally (+27.7% acceptance compared to software, *p* < 0.0001), major clinical impact (+4.3% vs mean clinical impact, *p* = 0.009) or minor clinical impact (−5.8% vs. mean, *p* = 0.0006), and resident prescriber (+4.4%, *p* = 0.007) (Table 2).

**TABLE 2:** Factors for acceptance of PIs, univariate and multivariate analysis.

Pharmacist interventions, all	Percentage of acceptance	Univariate percentage difference adjusted for pharmacist (%)	p univariate	Multivariate percentage difference (%)	p multivariate
Pharmacist					
Postgraduate pharmacist	1158/1426 (81.2%)	0 (Reference)	—	0 (Reference)	—
Pharmacy resident	1257/1504 (83.6%)	−3 (−21.2; 15.1)	0.73	−3 (−15.7; 9.8)	0.64
Prescriber					
Postgraduate physician	586/802 (73.1%)	0 (Reference)	—	0 (Reference)	—
**Medical resident**	**1829/2,128 (85.9%)**	**5.4 (2.2; 8.6)**	**0.0008**	**4.4 (1.2; 7.6)**	**0.007**
Patient gender					
Male	1190/1421 (83.7%)	0 (Reference)	—	0 (Reference)	—
Female	1225/1509 (81.2%)	1.9 (−0.5; 4.3)	0.13	2.5 (0.1; 4.9)	0.0412
Patient age					
≤59	716/773 (92.6%)	0 (Reference)	—	0 (Reference)	—
60–69	545/595 (91.6%)	−0.7 (−4.3; 2.8)	0.68	−0.4 (−3.8; 3.1)	0.82
70–79	465/569 (81.7%)	−3.7 (−7.4; 0)	0.0529	−2.6 (−6.2; 1)	0.16
80–89	485/682 (71.1%)	0.3 (−3.8; 4.4)	0.88	1.2 (−2.8; 5.2)	0.56
≥90 years	204/311 (65.6%)	−1.9 (−7; 3.3)	0.48	0.8 (−4.3; 5.9)	0.75
Problem encountered^a^					
Non-compliance with guidelines and contraindications	540/687 (78.6%)	−6.3 (−11.6; −0.9)	0.021	−4.4 (−9.9; 1)	0.11
Untreated indication	274/329 (83.3%)	0.4 (−5.4; 6.1)	0.90	4 (−3.3; 11.3)	0.29
Underdosing	323/392 (82.4%)	−4 (−9.6; 1.6)	0.16	−3.1 (−9.3; 3.2)	0.34
Overdosing	475/559 (85%)	0.8 (−4.6; 6.2)	0.77	1.1 (−4.6; 6.9)	0.70
Drug not indicated	145/203 (71.4%)	−4.1 (−10.4; 2.1)	0.20	−5.2 (−11.9; 1.5)	0.13
Drug interaction	120/135 (88.9%)	−2.6 (−9.6; 4.4)	0.47	−3 (−10.1; 4)	0.40
Adverse effect	49/57 (86%)	1.2 (−7.8; 10.3)	0.79	−5.4 (−14.3; 3.5)	0.23
Inappropriate route and/or administration	205/267 (76.8%)	−2.8 (−8.7; 3.2)	0.36	−2.5 (−9.5; 4.6)	0.49
Treatment not received	2/2 (100%)	15.8 (−25.1; 56.8)	0.45	17.7 (−22.1; 57.6)	0.38
Monitoring to be followed	282/299 (94.3%)	1.6 (−4.3; 7.6)	0.59	0.7 (−6.3; 7.8)	0.84
Type of Intervention					
Addition	346/413 (83.8%)	−1.6 (−5.4; 2.3)	0.43	−6.5 (−12.1; −0.9)	0.024
Discontinuation	412/497 (82.9%)	0.7 (−3; 4.4)	0.71	1 (−3.4; 5.5)	0.65
Substitution/Exchange	276/358 (77.1%)	−4.7 (−8.7; −0.7)	0.02	−1 (−5.3; 3.4)	0.66
Choice of route of administration	12/14 (85.7%)	8.8 (−6.1; 23.6)	0.25	5.1 (−10; 20.3)	0.51
Therapeutic follow-up	352/385 (91.4%)	1.2 (−2.8; 5.2)	0.55	2.4 (−2.9; 7.8)	0.37
Optimization of administration modalities	202/255 (79.2%)	−2.6 (−7; 1.8)	0.25	−0.1 (−5.7; 5.4)	0.96
Dosage adjustment	815/1008 (80.9%)	−1.8 (−5; 1.4)	0.26	−1 (−5.4; 3.3)	0.64
Method of contact					
Verbal	1545/1623 (95.2%)	0 (Reference)	—	0 (Reference)	—
Phone call	424/455 (93.2%)	−1.3 (−5.6; 3.1)	0.57	1.2 (−4.1; 6.4)	0.66
**Prescription assistance software**	**441/846 (52.1%)**	**−30 (−34.2; −25.8)**	**<0.0001**	**−27.7 (−32.1; −23.2)**	**<0.0001**
Paper	5/6 (83.3%)	−16.9 (−42.5; 8.8)	0.20	−15.1 (−41.4; 11.2)	0.26
Clinical impact on patient health					
None	38/41 (92.7%)	1.6 (−8.9; 12.1)	0.77	6.3 (−4.4; 16.9)	0.25
**Minor**	**395/570 (69.3%)**	**−6.7 (−10; −3.4)**	**<0.0001**	**−5.8 (−9.1; −2.5)**	**0.0006**
Moderate	1038/1317 (78.8%)	0 (Reference)	—	0 (Reference)	—
**Major**	**918/976 (94.1%)**	**5.1 (2; 8.3)**	**0.001**	**4.3 (1.1; 7.6)**	**0.009**
Vital	26/26 (100%)	9.1 (−3.7; 21.9)	0.16	7.6 (−4.9; 20.1)	0.23
Organizational impact					
Unfavorable	80/89 (89.9%)	1.7 (−5.3; 8.8)	0.63	−0.4 (−7.3; 6.6)	0.92
Nil	1682/2,130 (79%)	0 (Reference)		0 (Reference)	
Favorable	653/711 (91.8%)	3.6 (0.5; 6.8)	0.025	2.4 (−0.9; 5.6)	0.15
Economic impact					
Unfavorable	684/788 (86.8%)	0 (Reference)	—	0 (Reference)	—
Nil	928/1172 (79.2%)	−0.7 (−3.9; 2.5)	0.66	0.3 (−3.1; 3.8)	0.86
Favorable	803/970 (82.8%)	1.8 (−1.3; 5)	0.26	2.8 (−1.2; 6.9)	0.17
ATC class					
A Digestive tract and metabolism	300/409 (73.3%)	−1.9 (−6.9; 3.1)	0.46	−0.9 (−5.8; 4)	0.71
B Blood and blood-forming organs	407/508 (80.1%)	−2.7 (−7.5; 2.2)	0.28	−1.8 (−6.6; 3)	0.45
C Cardiovascular system	213/272 (78.3%)	−1.3 (−6.8; 4.1)	0.63	−4 (−9.3; 1.4)	0.15
D Dermatological drugs	6/8 (75%)	5.2 (−16.4; 26.8)	0.64	10.4 (−10.6; 31.4)	0.33
G Genitourinary system and sex hormones	23/33 (69.7%)	1.9 (−9.4; 13.2)	0.74	5.4 (−5.5; 16.4)	0.33
H Systemic hormones, excluding sex hormones	59/74 (79.7%)	1.9 (−6.1; 10)	0.63	3.2 (−4.6; 11.1)	0.42
J General anti-infectives for systemic use	820/887 (92.4%)	0.4 (−4.3; 5.2)	0.86	−2.1 (−6.9; 2.7)	0.39
L Antineoplastics and immunomodulators	64/68 (94.1%)	8.6 (−0.3; 17.5)	0.0589	1.6 (−7.2; 10.4)	0.72
M Muscle and skeleton	28/37 (75.7%)	−4.8 (−15.5; 5.9)	0.38	−6.1 (−16.5; 4.3)	0.25
N Nervous system	422/547 (77.1%)	−1 (−5.8; 3.9)	0.70	0.7 (−4.1; 5.4)	0.79
P Antiparasitic insecticides	5/7 (71.4%)	−11.8 (−34.9; 11.2)	0.31	−7.5 (−29.8; 14.8)	0.51
R Respiratory system	36/44 (81.8%)	7.6 (−2.3; 17.6)	0.13	4 (−5.7; 13.7)	0.42
S Sensory organs	12/14 (85.7%)	2.8 (−13.7; 19.4)	0.74	2.7 (−13.5; 18.8)	0.75
V Miscellaneous	18/19 (94.7%)	9.2 (−5.5; 23.9)	0.22	9.1 (−5.1; 23.2)	0.21
Z Drug with no code assigned	2/3 (66.7%)	−14.3 (−49.3; 20.7)	0.42	−14.6 (−48.7; 19.4)	0.40
PI at reconciliation					
No	2,133/2,586 (82.5%)	0 (Reference)	—	0 (Reference)	—
**Yes**	**282/344 (82%)**	**5.7 (1.5; 9.8)**	**0.008**	0.5 (−4.1; 5.1)	0.83
PI using Pharmaclass®					
No	2,284/2,786 (82%)	0 (Reference)	—	0 (Reference)	—
Yes	131/144 (91%)	−3.5 (−9.4; 2.5)	0.25	−6.2 (−13.6; 1.1)	0.095

a Use of contrasts −1/+1.

Parameters associated with *p* < 0.05 are shown in bold. The gray background highlights significant univariate associations alone or significance in both univariate and multivariate analyses.

In *post hoc* analysis, clinical impact on patient health was coded as a discrete quantitative variable to assess a linear trend in acceptance rate. In a linear model without adjustment or random pharmacist effect, moving to a higher clinical impact category was associated with an increase in acceptance rate of +10.7% (95% CI: 9.0–12.4%) on average. After adjustment for pharmacist (random effect), this increase was +5.0% (95% CI: 3.2–6.7%) per unit of impact. After adjustment for all the same covariates as in the main analysis, this increase was +4.0% (95% CI: 2.1–5.9%).

### Determination of Factors Associated With Accepted Pharmacist Interventions With Major or Vital Clinical Impact Using Available Modality Data (Available on Supplementary Data)

The clinical impact on patient health, when indicated, was major or vital in 34.2% (1,002/2,930) of all cases. Of these, 37.9% (696/1,834) of the PIs were performed in the wards and 66.8% (479/717) were performed by the pharmacists in the resuscitation unit. The clinical impact on patient health was major or vital for 72.2% (104/144) of Pharmaclass®-based PIs and 32.2% (898/2,786) of non-Pharmaclass®-based PIs.

Pharmacist interventions with major or vital clinical impact on patient health were accepted in 94.2% (944/1,002) of cases, while the acceptance rate was 76.3% (1,471/1,928) for PIs with less clinical impact.

An equivalent statistical analysis was performed to determine the factors associated with accepted PIs with major or vital clinical impact on patient health.

Based on the minimally adjusted statistical analysis, the factors associated with accepted PI with major or vital clinical impact on patient health were: PI performed orally (+16.3%, *p* < 0.0001 vs. with software), PI performed during reconciliation (+9.1%, *p* = 0.003), and nervous system drug (−10.5%, *p* = 0.003).

### Determination of Factors Associated With Accepted Pharmacist Interventions as a Function of Method Used to Contact Prescriber and Location of Pharmacist

Because oral PIs were statistically more accepted, a complementary analysis was performed to assess the impact of the analysis within the care units even though the two variables were highly correlated, making it necessary to interpret the analyses with caution. The main contribution of the analysis was based on two pharmacists who shared the activity. The difference, with no adjustment for either pharmacist or other variables, was estimated at +17.7% (95% CI: 14.9–20.5%, *p* < 0.0001) in favor of the within-the-ward medication review. In a minimally adjusted model (random pharmacist effect), the difference was estimated at −2.1% (95% CI: −6.8 to +2.6%, *p* = 0.38) for the within-the-ward vs. the within-the-pharmacy medication review. After adjustment for pharmacist and method of contact, this effect on acceptance rate was estimated at −3.1% (95% CI: −8.4% to +2.1%, *p* = 0.25). The within-the-ward medication review was therefore not significantly associated with an accepted PI. However, an orally transmitted PI was significantly more often accepted in the adjusted models. This can be explained by the fact that PIs made within the care units *via* the prescription assistance software (339) had a lower acceptance rate (−30.5%, 95% CI: −34.8% to −26.2%, *p* < 0.0001) compared with the 1,474 PIs done orally. These analyses were performed *post hoc*, guided by the strong discordance between the strong raw effect of the within-the-ward medication review (which was expected) and the lack of observation of such an effect in the main analysis model (which was unexpected).

## Discussion

The percentage of accepted PIs in this study (82.4%) is higher than the values published in the literature which were, for example, 56% in a French psychiatric hospital (Tambon et al., 2019), 67.2% in a French rheumatology department (Yailian et al., 2019), 74.1% in a French general hospital (Loustalot et al., 2019), but notably 67.8 and 67.6% in studies of the entire Act-IP© database between 2006 and 2009 (Bedouch et al., 2015) and between 2014 and 2018 (Videau et al., 2021). This acceptance rate is similar to that recently published by a Spanish team who reported an accepted PI rate of 74%, but included 15% of PIs whose status was not indicated (Garin et al., 2021). The percentage of acceptance in the present study may be due to the maturation of the activity over time and the implementation of several specialized clinical pharmacy projects, but biases related to the open nature of the study should not be overlooked.

During the 6 months of the study, more than 3,000 PIs were performed, and one-third were considered to have major or vital clinical impact for the patient (i.e., potentially reducing the risk of prolonged hospitalization, permanent disability, handicap, or need for intensive care). The clinical impact of these PIs is highly expected to affect the frequency of adverse drug reactions, especially as these reactions most often occur following new prescriptions. Additionally, they are avoidable in almost half of the cases (Davies et al., 2009; Elliott et al., 2021). Given the current average cost of a medication error in hospitals of $89.40 (Samp et al., 2014), this activity has potentially reduced health care expenditure by several hundred-thousand euros. This confirms the value of current clinical pharmacy activity and motivates its continued implementation and funding.

Despite the prioritization of pharmaceutical care in intensive care, geriatrics, orthopedic surgery, hematology, and oncology units, the profile of the type of PIs in the study is comparable to previously published data, regarding the leading problems encountered, i.e., non-adherence to guidelines and dosing errors (Bedouch et al., 2015; Loustalot et al., 2019). The main drug classes targeted by the PIs in this study were anti-infectives (30.3% of all PIs), nervous system drugs (18.7% of PIs), and blood and hematopoietic organ drugs (17.3% of PIs). These data are similar to those found in a French teaching hospital (Loustalot et al., 2019), in the Act-IP^©^ database (Bedouch et al., 2015), and in a Spanish study (Garin et al., 2021).

In a raw analysis, PIs were more often accepted when they were performed in the care units, which is in line with a French multicenter study conducted in 2010 (Bedouch et al., 2015). However, this association was not found in the adjusted statistical analyses and was replaced by the contact method used with the prescriber. Effectively, PIs performed using the prescription assistance software were significantly more often refused, including refusals within the departments. This adds precision to the type of environmental setting of the PI, as suggested by Bedouch et al. (2015): Direct contact should be favored as much as possible, ideally by grouping the PIs together in order to avoid task interruptions, and a real pharmacist-physician exchange should be sought. This oral transmission of the PI seems all the more important as the PI has a major clinical impact. It ensures that the information is transmitted to the prescriber and can be discussed. This also results in better documentation of the PIs with data not available in the patient record. Any pharmacist conducting a medication review should avoid one-way written communication in their daily practice.

The presence of pharmacists in the care units increases the number of PIs generated, since 62.6% of PIs were generated by the six pharmacists working in the care units during their activity in the ward. This greater number of PIs generated by pharmacists present in the wards can be explained by the significant amount of time dedicated to medication review, access to other sources of information through participation in information transmission, medical visits, more experience in the medication review process, and easier contact with prescribers (Benoit et al., 2007). Nevertheless, presence in the department is not sufficient in itself and procedures for pharmacist-physician pairing remain essential.

Previous works have suggested that the clinical impact on patient health of a PI facilitates its acceptance, but these studies had a small sample and did not use the CLEO scale (Zaal et al., 2020). The fact that PIs are significantly more often accepted when they have a major clinical impact on patient health should support the medical attention they generate. Prioritizing the activities that enable these PIs is a key issue that makes it possible to focus limited human resources in the pharmacy on activities with a high clinical impact on patient health, where the benefit to patients is greatest.

In a setting where medication reconciliation has proven its effectiveness in reducing hospital mortality (Bond and Raehl, 2007), it should be noted that this activity is significantly associated with accepted PIs in the analysis adjusted for pharmacist, but that this association is no longer significant in the model adjusted for all the modalities. This suggests that the acceptance generated by the reconciliation activity is linked to its ability to detect PIs with a significant clinical impact on patient health, and to its oral communication mode (Haute Autorité de Santé, 2018). Reconciliation can therefore indirectly be the source of these major and accepted PIs.

Other strategies, such as the assignment of pharmacists to high-risk care units and the preferential analysis of high-risk drugs, are also aimed at prioritizing activity and are associated with a higher frequency of PIs with a greater clinical impact on patient health. However, to ensure acceptance of these PIs, oral transmission should be preferred.

Prioritizing medication review according to the type of patients and drugs makes it possible to optimize available human resources and increase the rate of PIs issued, as has already been demonstrated (Allenet et al., 2009; Philippe et al., 2017; Gougeard et al., 2021). Mobile clinical pharmacy activity using MDSS tools could be a good compromise between proximity and prioritization of targeting.

Pharmacist interventions were more often transmitted and accepted by medical residents than by postgraduate physicians, which is attributed to a higher prescribing rate, greater availability, and openness to discussions about the appropriateness of prescriptions. Nevertheless, this difference disappeared for PIs rated major or vital, suggesting the recovery of the prescriber’s attention.

The parameters statistically correlated with an accepted PI are qualitatively and quantitatively different from those previously published from the extraction of the entire national PI registration database (Bedouch et al., 2015). In contrast to this study, Bedouch et al. found therapeutic classes (anti-infectives, antineoplastics, and immunomodulators), types of intervention (addition, discontinuation, exchange, or optimization of administration), pediatric or intensive care patients, and pharmacists integrated into care units as explanatory factors of PI acceptance (Bedouch et al., 2015). This difference could be explained by methodological advantages, such as adjustment for pharmacist and the addition of the CLEO scale in the multivariate model that further attenuates differences related to therapeutic classes, and type of intervention. The difference between these results may also be partly explained by this single-center approach, with a different profile of hospitalized patients [majority in the resuscitation unit in this study (24.5 vs. 1.7%)].

The variability in acceptance grouped by pharmacist is very high, represented by an inter-pharmacist standard deviation estimated at 12.6% after adjustment. In future works, it seems essential to us to collect new factors characterizing the pharmacist-physician relationship (respective experience, relationship of trust, existence of situational protocols) as well as to better characterize the moment of the exchange (time, respective arguments, modification of the PI during the exchange).

The study has several limitations. It is a retrospective, single center study and the documentation of the outcome was performed without independent reevaluation for all PIs. Pharmacists were instructed not to record PIs involving generic substitutions. As this is a daily activity among others, a lack of time to manually archive some PIs may also contribute to the non-exhaustiveness of the database (Bedouch et al., 2015; Videau et al., 2021). An inherent subjectivity is in the coding of PIs using the CLEO scale, even if the reliability of these elements is considered good (Vo et al., 2019). This subjectivity coupled with non-exhaustiveness may lead to an overestimation of the actual acceptance percentage or create additional pharmacist-dependent variability, partially mitigated by our quality procedures for medication review and independent verification for unreported outcomes.

The robustness of the study is due to the large number of PIs performed over the period, and the significant statistical contribution of the linear mixed effects model, which makes it possible to adjust for each pharmacist in univariate and multivariate analysis. The study also has the advantage of focusing on advanced clinical pharmacy practices such as pharmacy presence in the wards and the use of Pharmaclass® in daily practice. It highlights the need to develop a procedure for the transmission of PIs and the importance of targeting critical PIs, which are the two parameters clearly associated with better acceptance of PIs by physicians and can therefore provide the greatest benefit to the patient.

## Conclusion

This work shows that medication review makes it possible to identify a significant number of prescription optimizations. Improvement of the acceptance of these PIs by prescribers can be achieved by developing procedures for exchange (in particular the use two-way oral exchanges and avoiding the written one-way type by default) and by targeting interventions with a major clinical impact on patient health. These results suggest that it is important to work proactively on pharmaceutical care projects and to locate specialized clinical pharmacists as close as possible to prescribers potentially assisted by digital analysis tools. The development and sustainability of these activities represent a major public health issue.

## Data Availability

The original contributions presented in the study are included in the article/Supplementary Material, further inquiries can be directed to the corresponding author.

## Abbreviations

ATC, Anatomical Therapeutic Chemical classification; CLEO, clinical, economical and organizational; PI, pharmacist intervention; SFPC, French Society of Clinical Pharmacy.
